# Abstracts of the 33rd International Austrian Winter Symposium: Zell am See, Austria. 24-27 January 2018

**DOI:** 10.1186/s13550-017-0354-4

**Published:** 2018-01-23

**Authors:** K. Binzel, A. Adelaja, C. L. Wright, D. Scharre, J. Zhang, M. V. Knopp, E. J. Teoh, D. Bottomley, A. Scarsbrook, H. Payne, A. Afaq, J. Bomanji, N. van As, S. Chua, P. Hoskin, A. Chambers, G. J. Cook, V. S. Warbey, A. Chau, P. Ward, M. P. Miller, D. J. Stevens, L. Wilson, F. V. Gleeson, K. Scheidhauer, C. Seidl, M. Autenrieth, F. Bruchertseifer, C. Apostolidis, F. Kurtz, T. Horn, C. Pfob, M. Schwaiger, J. Gschwend, C. D’Alessandria, A. Morgenstern, C. Uprimny, A. Kroiss, C. Decristoforo, E. von Guggenberg, B. Nilica, W. Horninger, I. Virgolini, S. Rasul, N. Poetsch, A. Woehrer, M. Preusser, M. Mitterhauser, W. Wadsak, G. Widhalm, M. Mischkulnig, M. Hacker, T. Traub-Weidinger, C. L. Wright, K. Binzel, E. J. Wuthrick, E. D. Miller, P. Maniawski, J. Zhang, M. V. Knopp, Sebastijan Rep, Marko Hocevar, Janja Vaupotic, Urban Zdesar, Katja Zaletel, Luka Lezaic, S. Mairinger, Thomas Filip, M. Sauberer, S. Flunkert, T. Wanek, J. Stanek, N. Okamura, O. Langer, C. Kuntner, M. C. Fornito, R. Balzano, V. Di Martino, S. Cacciaguerra, G. Russo, D. Seifert, M. Kleinova, A. Cepa, J. Ralis, P. Hanc, O. Lebeda, M. Mosa, S. Vandenberghe, E. Mikhaylova, D. Borys, V. Viswanath, M. Stockhoff, N. Efthimiou, P. Caribe, R. Van Holen, J. S. Karp, K. Binzel, J. Zhang, C. L. Wright, P. Maniawski, M. V. Knopp, P. M. Haller, C. Farhan, E. Piackova, B. Jäger, P. Knoll, A. Kiss, B. K. Podesser, J. Wojta, K. Huber, S. Mirzaei, A. Traxl, K. Komposch, Elisabeth Glitzner, T. Wanek, S. Mairinger, M. Sibilia, O. Langer, M. C. Fornito, M. Russello, G. Russo, R. Balzano, S. Sorko, H. J. Gallowitsch, S. Kohlfuerst, S. Matschnig, M. Rieser, M. Sorschag, P. Lind, L. Ležaič, S. Rep, J. Žibert, N. Frelih, S. Šuštar, K. Binzel, A. Adelaja, C. L. Wright, D. Scharre, J. Zhang, M. V. Knopp, R. P. Baum, T. Langbein, A. Singh, M. Shahinfar, C. Schuchardt, G. F. Volk, H. R. Kulkarni, M. C. Fornito, S. Cacciaguerra, R. Balzano, G. V. Di Martino, G. Russo, W. H. Thomson, M. Kudlacek, M. Karik, C. Farhan, H. Rieger, W. Pokieser, K. Glaser, S. Mirzaei, V. Petz, C. Tugendsam, W. Buchinger, B. Schmoll-Hauer, I. P. Schenk, K. Rudolph, M. Krebs, G. Zettinig, V. Zoufal, T. Wanek, M. Krohn, S. Mairinger, J. Stanek, M. Sauberer, T. Filip, J. Pahnke, O. Langer, F. Weitzer, B. Pernthaler, S. Salamon, R. Aigner, P. Koranda, L. Henzlová, M. Kamínek, Mo. Váchalová, P. Bachleda, D. Summer, J. Garousi, M. Oroujeni, B. Mitran, K. G. Andersson, A. Vorobyeva, J.n Löfblom, A. Orlova, V. Tolmachev, C. Decristoforo, P. Kaeopookum, D. Summer, T. Orasch, B. Lechner, M. Petrik, Z. Novy, C. Rangger, H. Haas, C. Decristoforo

**Affiliations:** 1 Wright Center of Innovation in Biomedical Imaging, Department of Radiology, The Ohio State University Wexner Medical Center, Columbus, OH USA; 2 Department of Neurology, The Ohio State University Wexner Medical Center, Columbus, OH USA; 3 Departments of Radiology and Nuclear Medicine, Oxford University Hospitals NHS Foundation Trust, Oxford, UK; 4 The Leeds Teaching Hospitals NHS Trust, Leeds, UK; 5 University College London, London, UK; 6 The Royal Marsden NHS Foundation Trust, London, UK; 7 Mount Vernon Cancer Centre, London, UK; 8 King’s College London, London, UK; 9 Blue Earth Diagnostics, Oxford, UK; 10 TU München, Klinikum rechts der Isar, Nuklearmedizin, München, Germany; 11 TU München, Klinikum rechts der Isar, Urologie, München, Germany; 12 JRC, Institute for Transuranium, Karlsruhe, Germany; 13 Department of Nuclear Medicine, Medical University Innsbruck, Anichstrasse 32, 6020 Innsbruck, Austria; 14 Department of Biomedical Imaging and Image-guided Therapy, Division of Nuclear Medicine, Medical University of Vienna, Vienna, Austria; 15 Clinical Institute of Neurology, Medical University of Vienna, Vienna, Austria; 16 Clinical University of Internal Medicine I, Medical University of Vienna, Vienna, Austria; 17 CBmed GmbH, Center for Biomarker Research in Medicine, Graz, Austria; 18 Clinical University of Neuro-surgery, Medical University of Vienna, Vienna, Austria; 19 Wright Center of Innovation, The Ohio State University, Columbus, OH USA; 20 Radiation Oncology, The Ohio State University Wexner Medical Center, Columbus, OH USA; 21 Clinical Science, Philips Healthcare, Cleveland, OH USA; 22 Department of Nuclear Medicine, University Medical Centre Ljubljana, Ljubljana, Slovenia; 23 Department of Oncological Surgery, Oncology Institute Ljubljana, Ljubljana, Slovenia; 24 Jozef Stefan Institute Ljubljana, Ljubljana, Slovenia; 25 Institute of Occupational Safety Ljubljana, Ljubljana, Slovenia; 26 Biomedical Systems, Center for Health & Bioresources, AIT Austrian Institute of Technology GmbH, Seibersdorf, Austria; 27 Neuropharmacology, QPS Austria GmbH, Grambach, Austria; 28 Department of Clinical Pharmacology, Medical University of Vienna, Vienna, Austria; 29 Division of Pharmacology, Faculty of Medicine, Tohoku Medical and Pharmaceutical University, Sendai, Japan; 30 Nuclear Medicine Department PET/TC center Arnas Garibaldi Catania, Catania, Italy; 31 Pediatric Surgery Department Arnas Garibaldi Catania, Catania, Italy; 32 H. Pharmacy Department Arnas Garibaldi Catania, Catania, Italy; 33 Nuclear Physics Institute of the CAS, Rez, Czech Republic; 34 Charles university Faculty of Science Prague, Prague, Czech Republic; 35 MEDISIP research group, Ghent University, Ghent, Belgium; 36 UC Davis, Davis, CA USA; 37 Silesian University of Technology Gliwice, Gliwice, Poland; 38 PET instrumentation group, Department of Radiology, University of Pennsylvania, Philadelphia, Pennsylvania USA; 39 Wright Center of Innovation in Biomedical Imaging, Department of Radiology, The Ohio State University Wexner Medical Center, Columbus, Ohio USA; 40 Philips Healthcare, Cleveland, Ohio USA; 41 3rd Department of Medicine, Cardiology and Intensive Care Medicine, Chest Pain Unit, Wilhelminenhospital Vienna, Vienna, Austria; 42 Ludwig Boltzmann Cluster for Cardiovascular Research, Vienna, Austria; 43 Department of Nuclear Medicine with PET-Center, Wilhelminenhospital, Vienna, Austria; 44 Department of Biomedical Research, Medical University of Vienna, Vienna, Austria; 45 Department of Internal Medicine II, Division of Cardiology, Medical University of Vienna, Vienna, Austria; 46 Sigmund Freud University, Medical Faculty, Vienna, Austria; 47 Center for Health & Bioresources, Biomedical Systems, AIT Austrian Institute of Technology GmbH, Seibersdorf, Austria; 48 Institute of Cancer Research, Department of Medicine I, Comprehensive Cancer Center, Medical University of Vienna, Vienna, Austria; 49 Department of Clinical Pharmacology, Medical University of Vienna, Vienna, Austria; 50 Department of Biomedical Imaging and Image-Guided Therapy, Division of Nuclear Medicine, Medical University of Vienna, Vienna, Austria; 51 Nuclear Medicine Department PET/TC Center ARNAS Garibaldi, Catania, Italy; 52 Liver Unit ARNAS Garibaldi, Catania, Italy; 53 H.Pharmacy Department ARNAS Garibaldi, Catania, Italy; 54 Department of Nuclear Medicine and Endocrinology, PET/CT Center, Klinikum Klagenfurt, Klagenfurt, Austria; 55 Departments of Nuclear Medicine, University Medical Centre Ljubljana, Ljubljana, Slovenia; 56 Faculty of Health Sciences, University of Ljubljana, Ljubljana, Slovenia; 57 Wright Center of Innovation in Biomedical Imaging, Department of Radiology, The Ohio State University Wexner Medical Center, Columbus, OH USA; 58 Department of Neurology, The Ohio State University Wexner Medical Center, Columbus, OH USA; 59 Theranostics Center for Molecular Radiotherapy and Molecular ImagZentralklinik Bad Berka, Bad Berka, Germany; 60 Department of Otorhinolaryngology, Jena University Hospital, Jena, Germany; 61 Nuclear Medicine Department Arnas Garibaldi, Catania, Italy; 62 Pediatric Surgery Arnas Garibaldi, Catania, Italy; 63 Pharmacy H. Department Arnas Garibaldi, Catania, Italy; 64 Physics and Nuclear Medicine, City Hospital, Birmingham, UK; 65 Institute of Nuclear Medicine with PET-Center, Wilhelminenspital, Vienna, Austria; 66 Department of Viceral and General Surgery, Wilhelminenspital, Vienna, Austria; 67 Institute of Pathology and Microbiology, Wilhelminenspital, Vienna, Austria; 68 Schilddruesenpraxis Josefstadt, Vienna, Austria; 69 Schilddrueseninstitut Gleisdorf, Gleisdorf, Austria; 70 Department of Nuclear Medicine, Krankenanstalt Rudolfstiftung, Vienna, Austria; 71 Department of Nuclear Medicine, Sozialmedizinisches Zentrum Hietzing, Vienna, Austria; 72 Clinical Division of Endocrinology, Department of Medicine III, Medical University of Vienna, Vienna, Austria; 73 Center for Health & Bioresources, AIT Austrian Institute of Technology GmbH, Seibersdorf, Austria; 74 Department of Neuro-/Pathology, University of Oslo (UiO) and Oslo University Hospital (OUS), Oslo, Norway; 75 Department of Clinical Pharmacology, Medical University of Vienna, Vienna, Austria; 76 Meduni Graz, Univ. Klinik für Radiologie, Abteilung für Nuklearmedizin, Graz, Austria; 77 Department of Nuclear Medicine, University Hospital Olomouc and Palacky University, Olomouc, Czech Republic; 78 Department of Vascular and Transplantation Surgery, University Hospital Olomouc and Palacky University, Olomouc, Czech Republic; 79 Department of Nuclear Medicine, Medical University Innsbruck, Anichstrasse 35, A-6020 Innsbruck, Austria; 80 Institute of Immunology, Genetic and Pathology, Uppsala University, SE-75185 Uppsala, Sweden; 81 Division of Molecular Imaging, Department of Medicinal Chemistry, Uppsala University, SE-751 83 Uppsala, Sweden; 82 Division of Protein Technology, KTH Royal Institute of Technology, SE-10691 Stockholm, Sweden; 83 Department of Nuclear Medicine, Medical University Innsbruck, Innsbruck, Austria; 84 Division of Molecular Biology, Biocenter, Medical University Innsbruck, Innsbruck, Austria; 85 Faculty of Medicine and Dentistry, Institute of Molecular and Translation Medicine, Palacky University, Olomouc, Czech Republic; 86 Research and Development Division, Thailand Institute of Nuclear Technology, Nakhonnayok, Thailand

## OP01 Ultra-Fast Wholebody Oncologic FDG PET/CT: A new capability enabled by digital photon counting PET/CT technology

### K. Binzel^1^, A. Adelaja^1^, C. L. Wright^1^, D. Scharre^2^, J. Zhang^1^, M. V. Knopp^1^

#### ^1^Wright Center of Innovation in Biomedical Imaging, Department of Radiology, The Ohio State University Wexner Medical Center, Columbus, OH, USA; ^2^Department of Neurology, The Ohio State University Wexner Medical Center, Columbus, OH, USA

##### **Correspondence:** K. Binzel

**Aim:** To demonstrate the clinical capability of ultra-fast whole body PET acquisition enabled by digital photon counting PET (dPET) and to assess and compare its diagnostic and quantitative characteristics to current clinical PET acquisition.

**Methods:** Twenty-five patients scheduled for FDG whole body PET/CT were imaged using three separate acquisitions as part of intra-individual comparison study with a pre-commercial release dPET/CT (Vereos) and cPET/CT (Gemini, Philips, Cleveland). Standard cPET imaging was performed at ~75 min p.i. of ~ 450 MBq FDG with investigational dPET imaged at ~55 min p.i. The first dPET acquisition was performed using 90s/bed position, immediately followed by a 9s/bed position.

Acquisition which lead to average table times of ~15 and ~2 min. These were compared with standard-of-care 90s/bed position cPET. The 9s/bed dPET listmode data were reconstructed using a previously optimized methodology. All other aspects of image acquisition were kept identical. Three blinded reviewers evaluated the data sets regarding visual characteristics, diagnostic confidence and semi-quantitative readouts.

**Results:** Visual assessment scores were significantly higher for 90s/bed dPET whole body (p<0.01) with no difference between 9s/bed dPET and 90s/bed cPET. Quantitatively, the 9s/bed dPET images presented slightly increased background noise, however there was no significant impact on diagnostic confidence or SUV measures of FDG-avid lesions.

**Conclusion:** Next generation digital photon counting PET detector technology enables a new capability of Ultra-Fast (~2min) wholebody acquisition with comparable diagnostic confidence and quantitative precision to current generation cPET acquisitions taking 10 times longer. This allows for new PET workflow concepts, improved patient comfort, minimized patient motion and whole-body pseudo-dynamic imaging of tracer uptake.

**Reference**

1. Wright CL, Binzel K, Zhang J, Knopp MV. Advanced Functional Tumor Imaging and Precision Nuclear Medicine Enabled by Digital PET Technologies. Contrast Media & Molecular Imaging. 2017;2017:5260305. 10.1155/2017/5260305.

## OP02 The FALCON trial: Impact of ^18^F-fluciclovine PET/CT on clinical management choices for men with biochemically recurrent prostate cancer

### E. J. Teoh^1^, D. Bottomley^2^, A. Scarsbrook^2^, H. Payne^3^, A. Afaq^3^, J. Bomanji^3^, N. van As^4^, S. Chua^4^, P. Hoskin^5^, A. Chambers^5^, G. J. Cook^6^, V. S. Warbey^6^, A. Chau^7^, P. Ward^7^, M. P. Miller^7^, D.J. Stevens^7^, L. Wilson^7^, F. V. Gleeson^1^

#### ^1^Departments of Radiology and Nuclear Medicine, Oxford University Hospitals NHS Foundation Trust, Oxford, UK; ^2^The Leeds Teaching Hospitals NHS Trust, Leeds, UK; ^3^University College London, London, UK; ^4^The Royal Marsden NHS Foundation Trust, London, UK; ^5^Mount Vernon Cancer Centre, London, UK; ^6^King's College London, London, UK; ^7^Blue Earth Diagnostics, Oxford, UK

##### **Correspondence:** E. J. Teoh

**Aim:** Detection of the extent of local recurrence and of metastases in biochemical recurrence (BCR) of prostate cancer facilitates selection of appropriate treatment. The FALCON trial (NCT02578940) assessed the impact of 18F-fluciclovine PET/CT on the clinical management of men with BCR of prostate cancer following initial radical therapy.

**Methods:** Men being considered for curative-intent salvage therapy following first BCR were recruited at 6 UK sites. Management plans were documented prior to and following 18F-fluciclovine PET/CT imaging. Post-scan changes to treatment modality such as salvage radiotherapy [RT] to systemic therapy were classed as ‘major’, while changes within a modality (e.g. modified RT fields) were classed as ‘other’. A pre-planned interim analysis of the first 85 patients was conducted; recruitment was to be stopped for efficacy if the number of treatment changes was > 45 (52.9%; 97.5% CI: 40.3–62.3%), or for futility if ≤ 8 (9.4%, 97.5% CI: 3.6–18.9%).

**Results:** The 85 enrolled patients were a mean 4.8 y post-initial diagnosis, with a median age of 67 y and median PSA of 0.63ng/mL. Twelve (14.1%) had a Gleason score ≤ 6, 60 (70.6%) had a score of 7 and 13 (15.3%) had a score ≥ 8. Most (56; 65.9%) had previously undergone radical prostatectomy (RP), with 27 having received RT (± other therapy). The majority of those imaged (52; 61.2%) had a change in management (CIM) post-scan (see Table 1). Recruitment was subsequently stop as the pre-specified condition defining overwhelming efficacy was met.

**Conclusion:** This prospective trial shows 18F-fluciclovine PET/CT has substantial impact on clinical decisions for men with a first BCR of prostate cancer after curative-intent primary therapy.

**Table 1 (abstract OP02).** See text for description

Patients	N = 85
Detection rate:	n (%)
Patient	44/85 (51.8)
Prostate/bed	34/85 (40.0)
Extraprostatic	19/85 (22.4)
Pelvic lymph nodes	12/85 (14.1)
Bone	9/85 (10.6)
CIM: n (%)	52/85 (61.2)
Prior RP	24/52 (46.2)
Positive ^18^F-fluciclovine scan	41/52 (78.8)
Major CIM: n (%)	31/52 (59.6)
Salvage therapy to watchful waiting	13/52 (25.0)
Salvage therapy to systemic therapy	18/52 (34.6)
Other CIM: n (%)	21/52 (40.4)
Modified RT field	21/52 (40.4)

## OP03 Bi-213-anti-EGFR-MAb therapy of recurrent bladder cancer

### K. Scheidhauer^1^, C. Seidl^1^, M. Autenrieth^2^, F. Bruchertseifer^3^, C. Apostolidis^3^, F. Kurtz^2^, T. Horn^2^, C. Pfob^1^, M. Schwaiger^1^, J. Gschwend^2^, C. D’Alessandria^1^, A. Morgenstern^3^

#### ^1^TU München, Klinikum rechts der Isar, Nuklearmedizin, München, Germany; ^2^TU München, Klinikum rechts der Isar, Urologie, München, Germany; ^3^JRC, Institute for Transuranium, Karlsruhe, Germany

##### **Correspondence:** K. Scheidhauer

This abstract is not included here as it has already been published [1].

**Reference**

[1] K. Scheidhauer, C. Seidl, F. Bruchertseifer, et al. “Bi-213-anti-EGFR-MAb therapy of recurrent bladder cancer - a pilot study.” *Eur J Nucl Med Mol Imaging (2017) 44(Suppl 2):S130*.

## OP04 Early PET imaging of 68Ga-PSMA-11 PET/CT increases the detection rate of local recurrence in prostate cancer patients with biochemical recurrence

### C. Uprimny, A. Kroiss, C. Decristoforo, E. von Guggenberg, B. Nilica, W. Horninger, I. Virgolini

#### Department of Nuclear Medicine, Medical University Innsbruck, Austria; Anichstrasse 32, 6020 Innsbruck, Austria

##### **Correspondence:** C. Uprimny

This abstract is not included here as it has already been published [1].

**Reference**

[1] Uprimny C, Kroiss AS, Decristoforo C et al. “Additional value of early [68]Ga-PSMA-11 PET Imaging in the assessment of local recurrence in prostate cancer patients with biochemical recurrence.” *Eur J Nucl Med Mol Imaging (2017)44(Suppl 2):S404.*

## OP05 Gender Specific Analysis of 11C-Methionine PET Examination in Patients with Newly Diagnosed Gliomas

### S. Rasul^1^, N. Poetsch^1^, A. Woehrer^2^M. Preusser^3^, M. Mitterhauser^1^, W. Wadsak^14,^, G. Widhalm^5^, M. Mischkulnig^5^, M. Hacker^1^, T. Traub-Weidinger^1^

#### ^1^Department of Biomedical Imaging and Image-guided Therapy, Division of Nuclear Medicine, Medical University of Vienna, Vienna, Austria; ^2^Clinical Institute of Neurology, Medical University of Vienna, Vienna, Austria; ^3^Clinical University of Internal Medicine I, Medical University of Vienna, Vienna, Austria; ^4^CBmed GmbH, Center for Biomarker Research in Medicine, Graz, Austria; ^5^Clinical University of Neuro-surgery, Medical University of Vienna, Vienna, Austria

##### **Correspondence:** S. Rasul

**Aim:** L-[S-methyl- 11C]methionine (MET) PET is a highly sensitive and established method for glioma imaging. Little is known about gender specific aspects of glioma with respect to imaging characteristics. The aim of this study was to assess gender specific differences in distribution, localization as well as in diagnosis by using MET PET of newly diagnosed gliomas.

**Methods:** 160 glioma patients (45% female, mean age: 45, range 18 - 84 yrs.) were retrospectively analyzed. Gender specific differences based on clinical presentation at the time of the diagnosis, visual and semi-quantitative evaluation by means of tumor to background (T/N) ratio in pre-surgical MET PET, the isocitrate dehydrogenase 1-R132H mutational status (IDH1-R132H), and survival using Kaplan-Meier estimates and a Cox proportional hazard model were determined.

**Results:** There were no significant differences regarding clinical symptoms, tumor size, histology, and IDH1-R132H mutations between male and female patients at the time of diagnosis. Nevertheless, statistically significant differences were found for the surgical approach (tumor resection, open biopsy and stereotactic biopsy) as well as for visual PET analysis (focal, focal-areal, multifocal-areal, areal, and negative) between male and female studied patients (*P*< 0.01). The T/N ratio was significantly higher in male than in female patients (3.0 ± 1.5 vs. 2.5 ± 1.3, P= 0.03). Additionally, in comparison to female patients, male patients showed a higher number of occipital tumors (*P*= 0.02) and significantly lower median overall survival rate (76 vs. 112 months, P= 0.02).

**Conclusion:** Results of the study demonstrated gender specific differences in the localization of the tumor, MET PET analysis as well as in the surgical approaches and survival rate in patients with gliomas.

**Reference**

1. Rasmussen, B.K., Hansen, S., Laursen, R.J. et al. Epidemiology of glioma: clinical characteristics, symptoms, and predictors of glioma patients grade I–IV in the the Danish Neuro-Oncology Registry. J Neurooncol (2017). https://doi.org/10.1007/s11060-017-2607-5

## OP06 Assessing Post-Radioembolization Lung Shunt Fraction of Yttrium-90 Microspheres: A Clinical Feasibility Study Using Digital Photon Counting PET/CT

### C. L. Wright^1^, K. Binzel^1^, E. J. Wuthrick^2^, E. D. Miller^2^, P. Maniawski^3^, J. Zhang^1^, M. V. Knopp^1^

#### ^1^Wright Center of Innovation, The Ohio State University, Columbus, OH, USA; ^2^Radiation Oncology, The Ohio State University Wexner Medical Center, Columbus, OH, USA; ^3^Clinical Science, Philips Healthcare, Cleveland, OH, USA

##### **Correspondence:** C. L. Wright

**Aim:** Targeted radioembolization therapy with intra-arterially administered Yttrium-90 microspheres is routinely used for patients with unresectable hepatic malignancies and metastases. There remains an unmet clinical need for accurate imaging based assessment of Yttrium-90 (90Y) microsphere biodistribution in the lungs following radioembolization. At present, pre-radioembolization planning uses intra-arterial administration of 99mTc macroaggregated albumin (MAA) followed by quantitative scintigraphy to detect and measure extra-hepatic shunting from the liver to the lungs (i.e., lung shunt fraction or LSF). During radioembolization, catheter-directed microspheres may potentially pass through tumor-associated arteriovenous shunts and then become deposited within the pulmonary vasculature which can contribute to dose-dependent radiation pneumonitis [1]. Although scintigraphic bremsstrahlung imaging is routinely used to qualitatively verify the microsphere distribution within the treated liver, its utility in quantitatively assessing post-radioembolization LSF is greatly limited. It has been demonstrated that conventional PET imaging of the annihilation radiation generated by 90Y internal pair production can produce diagnostic images which can be further analyzed quantitatively [2]. Recently, digital photon counting PET/CT (dPET/CT) imaging for post-radioembolization assessment of 90Y microsphere biodistribution in the liver was demonstrated to be clinical feasible [3, 4]. Furthermore, preliminary results with dPET/CT demonstrates its capability for detecting discrete foci of ultra-low dose residual 90Y microspheres within post-radioembolization delivery systems (5-272 MBq). The aim of this study is to generate clinical feasibility data for dPET/CT estimation of 90Y microsphere LSF following radioembolization and compare it with the pre-therapy 99mTc MAA LSF.

**Methods:** Standard pre-therapy 99mTc MAA and post-therapy 90Y bremsstrahlung scintigraphic and SPECT/CT imaging was performed in 8 patients who underwent routine interventional radioembolization with 90Y glass microspheres. As part of an ongoing clinical trial, 90Y dPET/CT imaging of the lungs and liver was performed in each patient (4-50 h following radioembolization) using a total image acquisition time of 21 min. Intra-individual comparison of pre/post therapy SPECT/CT and post-therapy dPET/CT image quality, intrahepatic radioactivity distribution, pre-/post-therapy concordance, and volumetric assessment of intrahepatic radioactivity was performed using the Intellispace Portal workstation (Philips). The 99mTc MAA LSF was calculated using planar scintigraphy with regions of interest (ROI) placed around the lungs and liver on anterior and posterior planar imaging. Pre-therapy 99mTc MAA LSF was calculated using the geometric mean of the anterior and posterior counts of the lungs with respect to the anterior and posterior counts of the lungs plus liver. Digital PET/CT assessment of post-therapy 90Y microsphere LSF was performed using MIMVista (MIM Software). ROIs of the lungs and liver were again generating using a region-grow technique combined with thresholding. 90Y LSF with dPET/CT was calculated using the integral activity in the lungs with respect to the integral activity in the lungs plus liver.

**Results:** All patients had evaluable pre-therapy 99mTc MAA, post-therapy 90Y bremsstrahlung, and post-therapy 90Y dPET/CT images for qualitative assessment of intrahepatic radioactivity distribution. In all patients, 90Y dPET imaging enabled better image quality and increased 90Y-to-background contrast which improved qualitative and quantitative volumetric assessment of intrahepatic microsphere distribution when compared with bremsstrahlung SPECT. Post-therapy dPET/CT also improved assessment for concordance/discordance with pre-therapy MAA SPECT/CT when compared with bremsstrahlung SPECT/CT. Post-therapy 90Y bremsstrahlung SPECT consistently overestimated liver treatment volumes when compared with pre-therapy MAA SPECT and 90Y dPET. There were no instances of significant shunting of 90Y microspheres outside of the liver. No discrete foci of abnormally increased 90Y activity were detected in the lungs and dPET/CT estimation of 90Y LSF was consistently less than the pre-therapy MAA LSF.

**Conclusion:** To date, no quantitative PET measurement of the 90Y microsphere LSF following radioembolization has been reported and no data are currently available comparing the pre-therapy MAA LSF and the post-therapy 90Y microsphere LSF. One challenge is that there is no reference standard for estimating the LSF other than planar scintigraphic imaging of the chest and abdomen following the intra-arterial MAA administration. Given that 90Y microsphere dosage is based upon the pre-therapy 99mTc MAA LSF and that MAA particles are more variable in size (e.g., 10-90 microns) than 90Y microspheres (20-35 microns), quantitative estimation of the post-therapy 90Y microsphere LSF using dPET/CT approaches may have diagnostic and therapeutic impact by identifying patients with smaller or larger than anticipated LSFs. In particular, subsets of patients would be identified on post-therapy 90Y PET imaging that may be eligible for additional radioembolization therapy (i.e., patients with 50 Gy cumulative lung dose). Routine estimates of 90Y dPET LSF would also greatly increase our understanding of tumor-induced neovascularity and the degree of vascular shunting in various liver malignancies/metastases targeted by 90Y radioembolization therapy.

**Research Support:** This project was enabled by the Ohio Third Frontier Awards (TECH 09-028, TECH 10-012, TECH 13-060), Wright Center of Innovation in Biomedical Imaging Development Fund. Philips Healthcare provided the investigational pre-commercial release dPET/CT system. CLW is supported by American Cancer Society, Radiological Society of North America, and National Cancer Institute – Clinical Research Loan Repayment Program.

**References**

1. Wright, C.L., et al., Radiation pneumonitis following yttrium-90 radioembolization: case report and literature review. J Vasc Interv Radiol, 2012. 23(5): p. 669-74.

2. Wright, C.L., et al., Theranostic Imaging of Yttrium-90. Biomed Res Int, 2015. 2015: p. 481279.

3. Wright, C.L., et al., 90Y Digital PET/CT Imaging Following Radioembolization. Clin Nucl Med, 2016. 41(12): p. 975-976.

4. Wright, C.L., et al., Clinical feasibility of 90Y digital PET/CT for imaging microsphere biodistribution following radioembolization. Eur J Nucl Med Mol Imaging, 2017. 44(7): p. 1194-1197.

## OP07 Patient radiation exposure after parathyroid imaging in nuclear medicine

### Sebastijan Rep^1^, Marko Hocevar^2^, Janja Vaupotic^3^, Urban Zdesar^4^, Katja Zaletel^1^, Luka Lezaic^1^

#### ^1^Department of Nuclear Medicine, University Medical Centre Ljubljana, Ljubljana, Slovenia; ^2^Department of Oncological Surgery, Oncology Institute Ljubljana, Ljubljana, Slovenia; ^3^Jozef Stefan Institute Ljubljana, Ljubljana, Slovenia; ^4^Institute of Occupational Safety Ljubljana, Ljubljana, Slovenia

##### **Correspondence:** Sebastijan Rep

**Aim:** The aim of our study was to measure the ED and organ doses for conventional subtraction scintigraphy, dual-phase MIBI SPECT/CT and FCH dual-phase PET/CT.

**Methods:** Twenty-five patients referred for parathyroid imaging with a clinical indication of primary hyperparathyroidism underwent parathyroid subtraction scintigraphy and dual-phase SPECT/CT imaging with the addition of FCH PET/CT. Radiation exposure was calculated for administered activities of radiopharmaceuticals using ICRP weighting factors and for CT exposure at hybrid imaging using dose-length products and ImPACT CT Patient Dosimetry Calculator.

**Results:** The highest radiation exposure was caused by conventional parathyroid subtraction scintigraphy (7.27 mSv), followed by dual-phase MIBI SPECT/CT (6.81 mSv). The radiation exposure was lowest for dual-phase FCH PET/CT imaging (2.86 mSv). The added CT imaging for both hybrid approaches did not cause significant additional radiation exposure (1.41 mSv for MIBI SPECT/CT, additional 26.11 % to overall exposure; 0.86 mSv for FCH PET/CT, additional 43.51 % to overall exposure).

**Conclusion:** According to our results, emerging FCH PET/CT imaging techniques lower radiation exposure to patients in comparison to conventional scintigraphic imaging for HPG

**References**

1. Pallan S, Rahman MO, Khan AA. Diagnosis and management of primary hyperparathyroidism. BMJ (Clinical research ed.). 2012;344:e1013.

2. Lezaic L, Rep S, Sever MJ, Kocjan T, Hocevar M, Fettich J. ^18^F-Fluorocholine PET/CT for localization of hyperfunctioning parathyroid tissue in primary hyperparathyroidism: a pilot study. European journal of nuclear medicine and molecular imaging. 2014;41:2083-2089.

3. Cazaentre T, Clivaz F, Triponez F. False-positive result in 18F-fluorocholine PET/CT due to incidental and ectopic parathyroid hyperplasia. Clinical nuclear medicine. 2014;39:e328-e330.

4. Michaud L, Balogova S, Burgess A, Ohnona J, Huchet V, Kerrou K et al.. A pilot comparison of 18F-fluorocholine PET/CT, ultrasonography and 123I/99mTc-sestaMIBI dual-phase dual-isotope scintigraphyin the preoperative localization of hyperfunctioning parathyroid glands in primary or secondary hyperparathyroidism: influence of thyroid anomalies. Medicine. 2015;94:e1701.

5. Kluijfhout WP, Vorselaars WMCM, Vriens MR, BorelRinkes IHM, Valk GD, de Keizer B. Enabling minimal invasive parathyroidectomy for patients with primary hyperparathyroidism using Tc-99m-sestamibi SPECT-CT, ultrasound and first results of (18)F-fluorocholine PET-CT. European journal of radiology. 2015;84:1745-1751.

7. Larkin AM, Serulle Y, Wagner S, Noz ME, Friedman K. Quantifying the increase in radiation exposure associated with SPECT/CT compared to SPECT alone for routine nuclear medicine examinations. International journal of molecular imaging. 2011;2011:897202.

8. Montes C, Tamayo P, Hernandez J, Gomez-Caminero F, García S, Martín C et al.. Estimation of the total effective dose from low-dose CT scans and radiopharmaceutical administrations delivered to patients undergoing SPECT/CT explorations. Annals of nuclear medicine. 2013;27:610-617.

9. Radiation dose to patients from radiopharmaceuticals (addendum 2 to ICRP publication 53). Annals of the ICRP. 1998;28:1-126.

10. The 2007 Recommendations of the International Commission on Radiological Protection. ICRP publication 103. Annals of the ICRP. 2007;37:1-332.

11. Radiation dose to patients from radiopharmaceuticals (A fourth addendum to ICRP Publication 53). Annals of the ICRP. 2013;43:1-22.

## OP08 Sex differences in blood pharmacokinetics and metabolism of (S)-[18F]THK5117 and [18F]THK5351 in rats

### S. Mairinger^1^, Thomas Filip^1^, M. Sauberer^1^, S. Flunkert^2^, T. Wanek^1^, J. Stanek^1,3^, N. Okamura^4^, O. Langer^1,3^, C. Kuntner^1^

#### ^1^Biomedical Systems, Center for Health & Bioresources, AIT Austrian Institute of Technology GmbH, Seibersdorf, Austria; ^2^Neuropharmacology, QPS Austria GmbH, Grambach, Austria; ^3^Department of Clinical Pharmacology, Medical University of Vienna, Vienna, Austria; ^4^Division of Pharmacology, Faculty of Medicine, Tohoku Medical and Pharmaceutical University, Sendai, Japan

##### **Correspondence:** S. Mairinger

**Aim:** Tau deposition is one of the neuropathological hallmarks of Alzheimer’s disease (AD). Among the different developed radiotracers, the 2-arylquinoline derivatives (S)-[18F]THK5117 and [18F]THK5351 show high affinity for neurofibrillary tangles [1, 2]. In vivo quantification of tau binding is usually performed by calculating uptake ratios between tau-rich target regions and non-target regions in the brain. However, kinetic modelling has also been proposed as an alternative to determine tau binding. Whereas these radiotracers have been investigated in different mouse AD models, kinetic modelling is precluded in mice by their small blood volume. Due to their higher blood volume, rat models with tau pathology may thus offer the possibility to perform arterial blood sampling and kinetic modelling. To determine the feasibility of this approach, we measured blood pharmacokinetics and radiotracer metabolism in female and male rats.

**Methods:** Female and male rats (n=4-6) were cannulated via the femoral artery for continuous blood sampling. Blood sampling was performed every 5-6 sec for the first 3 min and then at 5, 10, 20, 30, 40, 50 and 60 min after (S)-[18F]THK5117 or [18F]THK5351 injection. After collection of the 60 min blood sample, animals were sacrificed and organs were excised. Blood from minute 5, 20 and 60 was processed to plasma. Radiolabelled metabolites in plasma, brain, liver and urine were analyzed by radio-thin-layer chromatography (radio-TLC).

**Results:** Plasma pharmacokinetics and metabolism were significantly different between female and male rats for both investigated radiotracers. (S)-[18F]THK5117 plasma clearance was faster in female (1.33±0.24 mL/h/kg BW) than in male (0.87±0.17 mL/h/kg BW) rats (*p*=0.003). For (S)-[18F]THK5117, the percentage of unmetabolized parent was different between both sexes (5 min: 63% (f) vs 78% (m), *p*=0.02; 20 min: 43% (f) vs 23% (m), p<0.0001; 60 min: 32% (f) vs 9% (m), p<0.00001). Similar observations were made for [18F]THK5351. Plasma clearance was faster in female compared to male rats (1.07±0.23 mL/h/kg BW vs 0.67±0.04 mL/h/kg BW, p=0.02) and the percentage of radiolabelled metabolites in plasma was also different (20 min: 50% (f) vs 19% (m), p=0.01; 60 min: 37% (f) vs 4% (m), *p*<0.0001). In the liver, 13% (f) vs 6% (m) unchanged parent was measured for (S)-[18F]THK5117 and 16% (f) vs 3% (m) for [18F]THK5351. In the brain, 91-96% and 84-90% of total radioactivity consisted of unmetabolized (S)-[18F]THK5117 and [18F]THK5351, respectively.

**Conclusion:** Our results show pronounced sex differences in blood pharmacokinetics and metabolism of [18F]THK5117 and [18F]THK5351 in rats. Female animals showed a faster plasma clearance of both radiotracers. These results underline the importance of investigating both sexes and also support the notion that individual input functions are needed for kinetic modelling analyses.

**References**

1 Okamura N, Furumoto S, Harada R, Tago T, Yoshikawa T, Fodero-Tavoletti M, et al. Novel 18F-labeled arylquinoline derivatives for noninvasive imaging of tau pathology in Alzheimer disease. J Nucl Med 2013;54:1420-7.

2. Harada R, Okamura N, Furumoto S, Furukawa K, Ishiki A, Tomita N, et al. 18F-THK5351: A Novel PET Radiotracer for Imaging Neurofibrillary Pathology in Alzheimer Disease. J Nucl Med 2016;57:208-14.

## OP09 High diagnostic value of a new renal tubular imaging agent “L – Ethylenedicysteine” in children dynamic scintigraphy

### M. C. Fornito^1^, R. Balzano^1^, V. Di Martino^1^, S. Cacciaguerra^2^, G. Russo^3^

#### ^1^Nuclear Medicine Department PET/TC center Arnas Garibaldi Catania, Catania, Italy; ^2^Pediatric Surgery Department Arnas Garibaldi Catania, Catania, Italy; ^3^H. Pharmacy Department Arnas Garibaldi Catania, Catania, Italy

##### **Correspondence:** M. C. Fornito

This abstract is not included here as it has already been published [1].

**Reference**

[1] M.C. Fornito, S. Russo, A. Ruggeri et al. “High diagnostic value of a new renal tubular imaging agent ‘ethylenedicysteine’ in children dynamic scintigraphy”. *Clin Transl Imaging (2017) 5 (Suppl 1):S110.*

## OP10 Peptides labelling with 64Cu and 68Ga on heterogeneous phase using a microfludic system

### D. Seifert^1^, M. Kleinova^1^, A. Cepa^1^, J. Ralis^1^, P. Hanc^1^, O. Lebeda^1^, M. Mosa^2^

#### ^1^Nuclear Physics Institute of the CAS, Rez, Czech Republic; ^2^Charles university Faculty of Science Prague, Prague, Czech Republic

##### **Correspondence:** D. Seifert

**Aim:** We present a new approach to solid phase peptide labeling on a microfluidic system based on the PMMA matrix with the c18t sorbent. The chip allows for in-situ labelling at elevated temperatures up to 100 °C. Labeling is performed in the three subsequent steps.

**Methods:** 1st step: The selected peptide precursor was loaded onto the microfluidic chip activated with 2 ml of water and 2 ml of EtOH and then flushed with 0.1 M MES mobile phase at pH 5.5. As a model peptide we chose DOTA-TOC that was injected in a 500 μl volume (12.5 μg) and deposited on the sorbent matrix (C18t, 20 mg in a volume of 100 μl).

2nd step: The microfluidic chip was heated up to 95 °C and a chosen radiometal was injected into the system (64Cu, 300 MBq, 1 MBq/μl in 0.1 MES buffer). The free radiometal was eluted to waste, while the chelated radiometal remained on the chip.

3rd step: The radiolabelled product was eluted from the system into the organic phase (injection of 100 μl of EtOH).

**Results:** More than 90 % of the 64Cu activity was absorbed on the chip. The radiolabelled product was then eluted with 100 μl of EtOH in amount of 180 MBq, i.e. in the overall yield of 60 % in ca 25 minutes. Radiochemical purity determined via HPLC was found to be > 98 %.

**Conclusion:** We tested a newly designed microfluidic chip system suitable for radiometal labellings. It was demonstrated on a particular example of DOTA-TOC and 64Cu that the system allows for rapid radiolabelling in three simple steps providing high radiochemical purity product. The chip may be easily operated in GMP-compliant mode and modified for various couples radiometal-precursor.

**References**

1. G. Pascali, P. Watts, and P. A. Salvadori, “Microfluidics in radiopharmaceutical chemistry,” Nuclear Medicine and Biology, vol. 40, no. 6, pp. 776–787, 2013.

2. M. Wang, W. Lin, K. Liu, M. Masterman-Smith, and C. K. Shen, “Microfluidics for positron emission tomography probe development,” Molecular Imaging, vol. 9, no. 4, pp. 175–191, 2010. View at Publisher · View at Google Scholar · View at Scopus;

3. Brian M. Zeglis and Jason S. Lewis, A practical guide to the construction of radiometallated bioconjugates for positron emission tomography Dalton Trans. 2011 June 21; 40(23): 6168–6195. 10.1039/c0dt01595d.

## OP11 PET20.0: Design of a monolithic Total Body PET with 2.00 mm spatial resolution and 20 x higher sensitivity

### S. Vandenberghe^1^, E. Mikhaylova^2^, D. Borys^3^, V. Viswanath^4^, M. Stockhoff^1^, N. Efthimiou^5^, P. Caribe^1^, R. Van Holen^1^, J. S. Karp^4^

#### ^1^MEDISIP research group, Ghent University, Ghent, Belgium; ^2^UC Davis, Davis, CA, USA; ^3^Silesian University of Technology Gliwice, Gliwice, Poland; ^4^PET instrumentation group, Department of Radiology, University of Pennsylvania, Philadelphia, Pennsylvania, USA; ^5^University of Hull, UK

##### **Correspondence:** S. Vandenberghe

**Aim:** The aim of this system is to simultaneously improve sensitivity and resolution with a major step compared to current state-of-the art PET while maintaining a reasonable cost for the total system. The first aim of this study is to show the potential of high resolution monolithic LYSO scintillator with SiPM readout for a compact Total Body (TB) PET design with only 3-4 times the detector material of a current PET-CT scanner. The second aim of this study is the design of a long axial TB-PET system and the determination of its sensitivity gains for different types of objects. The transverse diameter of the system is fixed at 65 cm (which fits nearly all patients) as we expect that the whole bore can be reconstructed by making use of the Depth-of-interaction capabilities of the monolithic detector.

**Methods:** This system is characterised with regards to point and line sensitivity for typical detector settings in current clinical PET systems. In this study the effects of changing the axial length of the scanner are studied in detail. Finally two options for imaging long objects (above 1 m) with the same compact system are described.

**Results:** The system results in excellent spatial resolution at the system level. The sensitivity gains for point sources and small objects (brain type) are limited and are comparable to the increase in cost of the system. For longer objects (1-2m) the gains go up to a factor 15-20 x. Scanners in the range of 70 cm-1m20 match well with the typical region of interest for PET imaging and have an optimal sensitivity gain.

**Conclusion:** PET20.0 combines high and uniform spatial resolution at the system with a major increase in sensitivity (close to a factor 20) for total body imaging. This is accomplished by increasing the detector material by a factor 3-4.

**Reference**

1. EANM, OP517, S. Vandenberghe, E. Mikhalyova, B. Brans, M. Defrise, T. Lahoutte, K. Muylle, R. Van Holen, D. R. Schaart, J. S. Karp, PET 20.0: a cost-efficient, 2mm spatial resolution Total Body PET with point sensitivity up to 22% and adaptive axial FOV of maximum 2.00m

## OP12 Clinical validation of EARL-compliant reconstruction of digital photon counting PET/CT: An intra-individual clinical comparison study to conventional photomultiplier tube-based PET/CT

### K. Binzel^1^, J. Zhang^1^, C. L. Wright^1^, P. Maniawski^2^, M. V. Knopp^1^

#### ^1^Wright Center of Innovation in Biomedical Imaging, Department of Radiology, The Ohio State University Wexner Medical Center, Columbus, Ohio, USA; ^2^Philips Healthcare, Cleveland, Ohio, USA

##### **Correspondence:** K. Binzel

**Aim:** Digital photon counting serves to improve both visual and quantitative performance of PET/CT through advances in system sensitivity, count statistics and time of flight timing resolution. Fitting this data to a normalization curve such as with EARL requires a reconstruction protocol secondary to the default high definition protocol. We validated a vendor suggested EARL-compliant protocol for clinical data sets acquired with digital photon counting PET via intra-individual comparison to images acquired on a conventional system.

**Methods:** 26 patients underwent PET/CT imaging on a conventional system (Philips Gemini TF 64, cPET) approximately 75 minutes post-injection of 480 MBq 18F-FDG and 55 minutes on a pre-commercial release digital photon counting PET/CT system (Philips Vereos, DPC dPET). Listmode data were reconstructed with default settings: cPET – 4mm isometric voxel, 3 iterations, 33 subsets; DPC dPET – 2mm isometric voxel, 3 iterations, 11 subsets, point spread function correction and 4.1 mm Gaussian filter applied. DPC dPET data were further reconstructed with an EARL-compliant protocol – 4mm isometric voxel, 3 iterations 13 subsets, 5mm Gaussian filter which was previously validated with phantom data (1). Regions of interest (ROIs) were placed over target lesions and in a variety of background tissues for quantitative comparison.

**Results:** The average SUVmax of target lesions for default DPC dPET reconstructions was 9.74. The cPET data presented a substantially lower average SUVmax at 6.47, as anticipated due to lower recovery coefficients. The EARL dPET reconstruction revealed quantitative values comparable to cPET with an average SUVmax of 6.49. The decrease was due to an increase in partial volume effects by use of a smaller reconstruction matrix/larger voxel size as well as the smoothing introduced by the use of a Gaussian filter alone. In background tissues, the SUVmean varied by less than 5% among all reconstruction settings.

**Conclusion:** The EARL-compliant reconstruction of DPC dPET clinical cases as applied to intra-individual comparison data lead to quantitative results which match conventional EARL PET/CT values. It was demonstrated and validated that while DPC dPET imaging has substantially improved recovery coefficients, secondary reconstructions can be performed to enable comparable quantification with conventional PET systems and existing databases or inclusion in clinical trials.

**Reference**

1. Koopman D, Groot Koerkamp M, Jager PL, et al. Digital PET compliance to EARL accreditation specifications. EJNMMI Physics. 2017;4:9. 10.1186/s40658-017-0176-5.

## PP01 Relationship of dual time point myocardial SPECT-CT with peak creatine kinase and cardiac troponin I levels

### P. M Haller^1,2^, C. Farhan^3^, E. Piackova^1,2^, B. Jäger^1,2,5^, P. Knoll^3^, A. Kiss^4^, B. K. Podesser^2,4^, J. Wojta^2,5^, K. Huber^1,2,6^, S. Mirzaei^3^

#### ^1^3rd Department of Medicine, Cardiology and Intensive Care Medicine, Chest Pain Unit, Wilhelminenhospital Vienna, Vienna, Austria; ^2^Ludwig Boltzmann Cluster for Cardiovascular Research, Vienna, Austria; ^3^Department of Nuclear Medicine with PET-Center, Wilhelminenhospital, Vienna, Austria; ^4^Department of Biomedical Research, Medical University of Vienna, Vienna, Austria; ^5^Department of Internal Medicine II, Division of Cardiology, Medical University of Vienna, Vienna, Austria; ^6^Sigmund Freud University, Medical Faculty, Vienna, Austria

##### **Correspondence:** C. Farhan

**Aim:** The aim of this sub-study of a randomized clinical trial was to evaluate the relationship of peak creatine kinase (CK) and peak sensitive cardiac Troponin I (sc-TnI) levels with perfusion defect and left ventricular ejection fraction (LVEF) by gated single-photon emission computed tomography myocardial perfusion imaging (SPECT-MPI) in patients with ST-elevation myocardial infarction (STEMI) undergoing primary percutaneous coronary intervention (pPCI) during the acute phase and one month later.

**Methods:** Patients who were admitted with STEMI within 8 hours of symptom onset underwent gated SPECT-MPI acquisition (SPECT-LDCT, Siemens Symbia T6, Erlangen) within 24 hours (timepoint (TP) 1) after pPCI and one month later (TP 2). The MPI SPECT data were reconstructed and analyzed using 4DM-SPECT software. The infarct size was defined as percentage of the total left ventricular myocardium (%LV). Serial CK (U/L) und sc-TnI (μg/L) levels were evaluated at presentation and 2, 6, 12, 24 and 48 hours after pPCI, respectively. Both, peak NT-proBNP (ng/L) values and LVEF in ECHO were assessed during day 2 and 5 after admission.

**Results:** Twenty-four consecutive patients (mean age 60±12 years, 8 females) were included in this analysis. All patients had TIMI 3 flow in the culprit vessel after pPCI. In total 19 patients underwent SPECT on TP1 and 17 on TP2. The median perfusion defect was significantly larger on TP1 compared to TP2 (34%LV (IQR 16-54) vs. 16%LV (IQR 4-22), p=0.008). We found significant correlations for peak sc-TnI and peak CK levels with the perfusion defect assessed on TP1 (r=0.5, p=0.031; r=0.51, p=0.026; respectively) and on TP2 (r=0.88, p<0.001; r=0.92, p<0.001, respectively). Nt-proBNP values did not significantly correlate with the perfusion defect on either TP1 or TP2 (r=0.486, p=0.056; r=0.329, p=0.297), respectively. However, we found good correlations for LVEF between ECHO and SPECT assessment at TP1 and TP2 (r=0.7, p=0.005 and r=0.68, p=0.007).

**Conclusion:** The results of this pilot study demonstrate a significant correlation between infarct size assessed by use of SPECT-MPI and peak values of the more specific cardiac biomarkers CK and sc-TnI. This correlation was much higher one month compared to 24 hours after pPCI. LVEF calculated with SPECT-MPI showed good correlation to echocardiography irrespective of the TP. However, Nt-proBNP, a marker of left ventricular function, was not correlated with infarct size, which might be explained by stunning of potentially vital myocardium.

**References**

1. Byrne RA, Ndrepepa G, Braun S, Tiroch K, Mehilli J, Schulz S, Schömig A, Kastrati A. Peak cardiac troponin-T level, scintigraphic myocardial infarct size and one-year prognosis in patients undergoing primary percutaneous coronary intervention for acute myocardial infarction. Am J Cardiol. 2010 Nov 1;106(9):1212-7.

2. Arruda-Olson AM, Roger VL, Jaffe AS, Hodge DO, Gibbons RJ, Miller TD. Troponin T levels and infarct size by SPECT myocardial perfusion imaging. JACC Cardiovasc Imaging. 2011 May;4(5):523-33.

## PP02 Visualization of Abcg2 transport activity in the liver with [11C]erlotinib and positron emission tomography

### A. Traxl^1^, K. Komposch^2^, Elisabeth Glitzner^2^, T. Wanek^1^, S. Mairinger^1^, M. Sibilia^2^, O. Langer^1,3,4^

#### ^1^Center for Health & Bioresources, Biomedical Systems, AIT Austrian Institute of Technology GmbH, Seibersdorf, Austria; ^2^Institute of Cancer Research, Department of Medicine I, Comprehensive Cancer Center, Medical University of Vienna, Vienna, Austria; ^3^Department of Clinical Pharmacology, Medical University of Vienna, Vienna, Austria; ^4^Department of Biomedical Imaging and Image-Guided Therapy, Division of Nuclear Medicine, Medical University of Vienna, Vienna, Austria

##### **Correspondence:** A. Traxl

**Aim:** Breast cancer resistance protein (Abcg2) is an ATP-binding cassette transporter and expressed in the canalicular (bile-facing) membrane of hepatocytes, where it promotes hepatobiliary excretion of diverse drugs and their metabolites. Inter-individual variability in hepatic Abcg2 transport activity may affect the safety and efficacy of drugs excreted via hepatic Abcg2 from the body. We have shown before that the radiolabeled tyrosine kinase inhibitor [11C]erlotinib undergoes in mice Abcg2-mediated hepatobiliary excretion, suggesting an utility to measure the transport activity of Abcg2 in the liver [1]. Aim of this study was to investigate if [11C]erlotinib PET is sensitive enough to measure a reduction of Abcg2 expression in the liver using a transgenic mouse model, in which the epidermal growth factor receptor (EGFR) was cell-type specifically deleted in hepatocytes of the liver leading to a down-regulation of Abcg2.

**Methods:** Healthy male EGFR∆hep mice (lacking EGFR in hepatocytes of the liver, n = 5) and EGFRfl/fl mice (having normal EGFR expression in hepatocytes of the liver, n = 6) underwent an MR scan followed by a 90-min dynamic PET scan after intravenous injection of [11C]erlotinib. Concentration-time curves were derived for different organs from the co-registered PET/MR images and the area under the curve (AUC) from 0-90 min was calculated. Integration plot analysis was used to estimate the rate constant for transfer of radioactivity from the liver via bile into the intestine (kbile). Abcg2 protein expression levels in liver tissue collected at the end of PET imaging were determined with Western blot analysis.

**Results:** Western blot analysis revealed a significant reduction (-65%) of hepatic Abcg2 protein expression levels in EGFR∆hep mice relative to EGFRfl/fl mice. Intestinal AUC and kbile values were significantly lower in EGFR∆hep as compared with EGFRfl/fl mice (intestine AUC, EGFRfl/fl: 1,340 ± 368 %ID/g*min, EGFR∆hep: 866 ± 86 %ID/g*min; kbile, EGFRfl/fl: 0.018 ± 0.003 min-1, EGFR∆hep: 0.006 ± 0.002 min-1).

**Conclusion:** Our findings suggest that [11C]erlotinib PET is suitable to measure a reduction in Abcg2 expression in the liver. Thus, [11C]erlotinib PET may be used to study the influence of liver disease, genetic polymorphisms or drugs on hepatic Abcg2 transport activity.

**Reference**

1. Traxl et al. J Nucl Med. 2015 Dec;56(12):1930-6.

## PP03 Role of 18F-FDG PET in differentiation of Portal Malignant Thrombus from Bland Thrombus in patient with Hepatocellular carcinoma (HCC): a case report

### M. C.Fornito^1^, M. Russello^2^, G. Russo^3^, R. Balzano^1^

#### ^1^Nuclear Medicine Department PET/TC Center ARNAS Garibaldi, Catania, Italy; ^2^Liver Unit ARNAS Garibaldi, Catania, Italy; ^3^H.Pharmacy Department ARNAS Garibaldi, Catania, Italy

##### **Correspondence:** M. C. Fornito

**Aim:** Portal neoplastic thrombus, present in 6.5%-44% of patients with HCC, represents an important determinant of tumor staging and prognosis and influences treatment selection.HCC invasion into the portal vein renders a patient unsuitable for aggressive treatment such as surgical resection, liver transplantation or chemoembolization, due the high incidence of tumor recurrence. The standard for characterizing portal vein thrombus is histopathologic examination; in clinical practice others diagnostic imaging, clinical and laboratory findings are often utilized for discrimination. We report a case of a 63 yrs male affected by HBV cirrhosis and biopsy-proven multifocal HCC treated ten months with Sorafenib.He showed a good response to the disappearance of liver lesion and reduction of AFP value but the CT found a portal thrombus suspected as neoplastic that would make the patient unsuitable to the programmed liver transplantation.Diagnostic imaging as US, enhanced US and CT were not diagnostic. No MRI was performed for the presence of foreign metallic bullet. The patient was referred to 18F-FDG PET/CT for an accurate differentiation of bland from neoplastic thrombus, basing on the data published and considering the presence of a highly metabolic pattern in malignant vein thrombosis.

**Methods:** Data acquisitions by an integrated PET/CT system (Philips Gemini XLC) were performed within 60 min after 216 MBq of 18F-FDG. A late liver image was registered.

**Results:** The 18F-FDG PET/CT showed a high uptake in the main portal vein (SUV max 3.97) which increased in the late acquisition.Liver SUV was 2.45 and aortic SUV was 1.78. It is reported that in PET/CT thrombi were considered malignant if the SUV is greater than normal liver and/or the uptake is greater than that of the descending aorta in the same axial slice with an optimal cutoff value of SUV max 2.3-3.6. In our case the SUV value was consistent with malignant vein thrombosis. Considering that shrinkage of the thrombus and/or recanalization of the vessels was a definitive evidence of the benign nature of the thrombosis, the patient was followed-up monthly by US, CT and AFP value. After four months the enhanced CT showed an enlargement of the thrombus and parenchymal infiltration consistent with malignancy.

**Conclusion:** 18F-FDG PET/CT may be helpful in discriminating between benign and malignant portal vein thrombi. Patients may benefit from 18F-FDG PET/CT when portal vein thrombi cannot be diagnosed exactly by US, CT or when MRI isn’t feasible.

**Consent to publish:** Consent to publish was obtained from the patient involved in this study

**References**

1. Surasi DS, O'Malley JP, Bhambhvani P, J Nucl Med Technol 2015 Sep;43(3):229-30

2. Hu S,Zhang J, Cheng C, Liu Q, Sun G, Zuo C. Abdom Imaging 2014 Dec; 39(6):1221-7

## PP04 Lenvatinib-therapy for patients with radioiodine-refractory differentiated thyroid cancer – our experiences with Lenvima® at the Department of Nuclear Medicine and Endocrinology in Klagenfurt, Austria

### S. Sorko, H. J. Gallowitsch, S. Kohlfuerst, S. Matschnig, M. Rieser, M. Sorschag, P. Lind

#### Department of Nuclear Medicine and Endocrinology, PET/CT Center, Klinikum Klagenfurt, Klagenfurt, Austria

##### **Correspondence:** S. Sorko

This abstract is not included here as it has already been published [1].

**Reference**

[1] Stefan Sorko, Hans-Juergen Gallowitsch, Susanne Kohlfuerst, et al. “Lenvatinib-therapy for patients with radioiodine-refractory differentiated thyroid cancer - our experiences with Lenvima® at the department of nuclear medicine and endocrinology in Klagenfurt, Austria*.” Eur Thyroid J 2017;6(suppl 1):102.*

## PP05 The influence of attenuation correction and acquisition time on the SPECT image quality

### L. Ležaič^1^ S. Rep^1^, J. Žibert^2^, N. Frelih^1^, S. Šuštar^1^

#### ^1^Departments of Nuclear Medicine, University Medical Centre Ljubljana, Ljubljana, Slovenia; ^2^Faculty of Health Sciences, University of Ljubljana, Ljubljana, Slovenia

##### **Correspondence:** S. Šuštar

**Aim:** The aim of this article is to find out how the attenuation correction in the case of the SPECT/CT imaging influences the quality of the image, and also the effect of different acquisition time.

**Methods:** The Phantom NEMA IEC BodyPhantom was filled with the isotope technetium-99m. Eight images were captured, each with a different ratio of the specific activity between the phantom background and the spheres and also different acquisition time. The images were reconstructed in the program called Oasis by three different reconstructions: the filtered back projection, the non-corrected iterative reconstruction, and with the attenuation correction using the CT. The number of counts in the background and in all six spheres was measured. This was followed by the comparison of the contrast in images that were reconstructed using different methods. A descriptive statistic and repeated ANOVA measure were conducted. The Wilcoxon signed-rank test was carried out in Matlab.

**Results:** On the images that were processed by the filtered back projection or the iterative reconstruction, the background is not homogeneous. On the images that were corrected using the CT, the number of counts is evenly spread across the entire background of the phantom, thus making the background homogeneous. The statistical analysis showed that p < 0.001, meaning that, statistically, contrast is typically divergent among the different methods of reconstruction.

**Conclusion:** It was discovered that the increase in the number of counts, and consequently the image contrast, is proportional to the size of the sphere, and to the increased ratio of activity between the background and the sphere with all three types of reconstruction. The CT-AC images have the best contrast; images with iterative reconstruction are second best; images processed by the filtered back projection are third. The images reconstructed with the CT-AC have the least negative contrast, while the negative contrast in the images with iterative reconstruction and FBP images is far more prominent. Also increased imaging time results in increased count number and better image quality. Because the CT-AC images are of better quality than the non-corrected images, it is recommended that the CT-AC be used for all tests; however due to the removal of artefacts caused by attenuation correction, it is also important to examine the non-corrected images every time.

**References**

1. Grosser O S, Kupitz D, Ruf J et al. (2015). Optimization of SPECT-CT Hybrid Imaging Using Iterative Image Reconstruction for Low-Dose CT: A Phantom Study. 10.1371/journal.pone.0138658.

2. James A Patton, Timothy G Turkington (2008). SPECT/CT Physical Principles and Attenuation Correction. J Nucl Med Technol 36: 1–10.

3. Magdy M K Ed. (2011). Basic Sciences of Nuclear Medicine. London: Springer.

## PP06 Optimization of high definition digital PET/CT reconstruction for neurologic imaging

### K. Binzel^1^, A. Adelaja^1^, C. L. Wright^1^, D. Scharre^2^, J. Zhang^1^, M. V. Knopp^1^

#### ^1^Wright Center of Innovation in Biomedical Imaging, Department of Radiology, The Ohio State University Wexner Medical Center, Columbus, OH, USA; ^2^Department of Neurology, The Ohio State University Wexner Medical Center, Columbus, OH, USA

##### **Correspondence:** K. Binzel

**Aim:** Digital photon counting allows for higher definition image reconstruction than on conventional PET systems due to increased system sensitivity, time of flight timing resolution, and count densities (1). For neurologic applications, these improvements may greatly increase the utility and accuracy of PET imaging. We validated and optimized high definition image reconstruction methodologies using the Hoffman brain phantom.

**Methods:** A Hoffman brain phantom was filled with 52 MBq 18F-FDG and imaged on a pre-commercial release digital photon counting PET/CT (Philips Vereos). Five minute acquisitions were repeated over several runs using the dedicated brain 256 mm field of view (FOV) and the whole body 576 mm FOV. Listmode data from each acquisition was reconstructed using the high definition (HD) 2x2x2 mm voxel matrix. Reconstructions were performed with 3 iterations and a range of subsets, 21, 17, 13, and 9. Additionally, the use of system point spread function (PSF) correction and/or a Gaussian filter were enabled. Regions of interest (ROIs) were placed in 10 unique regions of the phantom for quantitative assessment. Blinded reader reviews were performed to assess image quality.

**Results:** We found that the optimal reconstruction settings for each FOV were distinct. For the 576 mm FOV the use of PSF alone, no Gaussian filter, gave the most accurate quantitative results. The addition of the Gaussian filter resulted in underestimation of activity concentrations. The average recovery coefficients (RCs) of all ROIs were very similar among reconstructions with different numbers of subsets. The average RCs were 0.97, 0.95, 0.94, and 0.92 for the 21, 17, 13, and 9 subsets reconstructions, respectively. Blinded review conveyed that the 13 subset images were most preferable with regard to contrast and image noise. Thus the HD reconstruction with PSF only using 3 iterations and 13 subsets was optimal for the whole body FOV acquisitions. For the 256 mm FOV a Gaussian filter was used in reconstruction as the PSF alone lead to overestimation of activity concentrations. As with the whole body FOV, each subset setting gave similar quantitative results, average RCs were 1.02, 1.01, 0.98, and 0.95 for the 21, 17, 13, and 9 subset reconstructions. Image review again showed that fewer subsets were preferred, with the 9 subset reconstruction now being most ideal. For acquisitions with the dedicated brain FOV, HD reconstruction with both PSF correction and a Gaussian filter using 3 iterations with 9 subsets is optimal for imaging with FDG.

**Conclusion:** Optimization of Neuro-PET reconstruction settings revealed that these setting must be tailored to acquisition characteristics, namely the chosen field of view. Phantom validation demonstrated that high quantitative accuracy and excellent image quality is readily achieved for neurologic imaging on next-generation digital PET systems when using either a dedicated brain field of view or the wider whole body field of view.

**Reference**

1. Wright CL, Binzel K, Zhang J, Knopp MV. Advanced Functional Tumor Imaging and Precision Nuclear Medicine Enabled by Digital PET Technologies. Contrast Media & Molecular Imaging. 2017;2017:5260305. 10.1155/2017/5260305.

## PP07 Injection of botulinum toxin for preventing salivary gland toxicity after PSMA-Radio-Ligand-Therapy - an empiric proof of concept for the future

### R. P. Baum^1#^, T. Langbein^1#^, A. Singh^1^, M. Shahinfar^1^, C. Schuchardt^1^, G. F. Volk^2^, H. R. Kulkarni^1^

#### ^1^Theranostics Center for Molecular Radiotherapy and Molecular ImagZentralklinik Bad Berka, Bad Berka, Germany; ^2^Department of Otorhinolaryngology, Jena University Hospital, Jena, Germany

##### **Correspondence:** R. P. Baum; T. Langbein

^#^authorship equally shared/equally contributed

**Aim:** The potential dose-limiting toxicity - especially when using alpha-emitters like Ac-225- or Bi-213, remains an unresolved issue in PSMA radioligand therapy (PRLT) of metastatic castration-resistant prostate cancer (mCRPC) due to the normal biodistribution of PSMA-radioligands in the salivary as well as in the lacrimal glands. Experience from over decades in external beam radiotherapy (EBRT) of the head and neck region demonstrates that severe xerostomia could become a life-quality-limiting factor. Since recent studies suggest a more PSMA-independent uptake in the salivary and lacrimal glands (1), suppressing the gland metabolism appears to be a promising method to achieve a lower uptake. Because of its safety and feasibility, injection of botulinum toxin into the salivary glands is commonly used to treat severe sialorrhoea (even in children) and has already been investigated as a potential radioprotective agent in EBRT (2).

**Methods:** A 63-year-old patient with mCRPC received multifocal ultrasound-guided injections of botulinum toxin A, totally 80 units, into his right parotid gland after a detailed informed consent. A dynamic salivary gland scintigraphy (SGS) using Tc-99m pertechnetate was performed before and after 70 h. At 45 days p.i., SGS as well as Ga-68-PSMA-PET/CT were performed. Standardized uptake values (SUV) of the right and left parotid glands were compared to the baseline PET/CT study. The patient underwent regular clinical follow up.

**Results:** Ga-68-PSMA-PET/CT performed 45 days after the botulinum toxin injection showed a heterogeneous, but highly significant reduction of the radioligand uptake by up to 60 % (SUVmean) in the injected right-sided parotid gland as compared to the left one. There was a drop of the SUVmean in the right parotid gland by up to 64 %, whereas no significant change in uptake of the left parotid gland was noted, when compared to the baseline PET/CT. The SGS also revealed a distinct decline in the peak uptake of Tc-99m pertechnetate in the injected parotid gland. The patient didn’t report any adverse effects of the injections throughout the follow-up period of now up to 2 months.

**Conclusion:** Intraparenchymal botulinum toxin injection impressively decreases the uptake of PSMA radioligands in the salivary glands. Therefore this approach (named 4B protection), which is described here for the very first time and was pioneered by our group, could be a significant breakthrough for salivary gland protection under PSMA radioligand therapy.

**References**

1. Kratochwil C, Bruchertseifer F, Rathke H, et al. Targeted alpha therapy of mCRPC with 225Actinium-PSMA-617: dosimetry estimate and empirical dose finding. J Nucl Med. April 13, 2017 [Epub ahead of print].

2. Teymoortash A, Pfestroff A, Wittig A, Franke N, Hoch S, Harnisch S, et al. Safety and Efficacy of Botulinum Toxin to Preserve Gland Function after Radiotherapy in Patients with Head and Neck Cancer: A Prospective, Randomized, Placebo-Controlled, Double-Blinded Phase I Clinical Trial. PLoS One. 2016 Mar 18;11(3) eCollection 2016

## PP08 Assessment of renal scars and differential renal function (DRF) in children with ureteral reflux: Tc 99m L-ethylenedicysteine dynamic scan vs Tc 99m DMSA static scan

### M. C. Fornito^1^, S. Cacciaguerra^2^, R. Balzano^1^, G. V. Di Martino^1^, G. Russo^3^

#### ^1^Nuclear Medicine Department Arnas Garibaldi, Catania, Italy; ^2^Pediatric Surgery Arnas Garibaldi, Catania, Italy; ^3^Pharmacy H. Department Arnas Garibaldi, Catania, Italy

##### **Correspondence:** M. C. Fornito

**Aim:** 99mTc-dimercaptosuccinic acid (DMSA) is an agent used for the diagnosis and follow-up of renal scarring in children with ureteral reflux detecting scars and calculating the differential renal function (DRF). DMSA has some disadvantages such as relatively higher radiation dose and time consumption. Recently a new radiopharmaceutical Tc-99m-ethylenedicysteine (EC) has been developed for dynamic scintigraphy and to measure renal function as an alternative to OIH and Tc-99m-MAG3, with comparable characteristics. EC provides high-quality images and low radiation dose. The purpose of this study was to evaluate the effectiveness of EC scan compared with DMSA in the detection of cortical lesions and DRF in order to perform only one scan with the advantage in terms of economy and health care.

**Methods:** 20 children 0.2-2 years with ureteral reflux (III and IV) were studied with DMSA and EC performed within 20-45 days. DMSA was acquired 3-4 hours after i.v. injection (EAMN paediatric guidelines) with 8 views (A/P, OP, PA and LL) by Philips Bright View XCT, LEHR collimator, 256 matrix, 300 kcounts/view. EC was performed with both detectors using the same equipment and recording dynamic anterior/posterior images after bolus in a 64 matrix for 30 minutes. First 180sec. images were summed in statics images in AP views. Perirenal ROI’s were drawn automatically for uptake and background and DRF was calculated. All images were also evaluated visually to define parenchymal abnormalities. All patients underwent to external dose rate measurements to evaluate the total decay constant (physical + biological).

**Results:** EC provided high-quality images even in newborns with a low/negligible extra-renal clearance, low liver and intestinal activity and high kidney-to-background ratio similar to DMSA, good delineation of the kidneys and detection of renal scars. DFR calculated and compared for both agents showed a close correlation (r =0.979) between values obtained by the two different methods.The qualitative analysis from DMSA and EC revealed 22 and 18 focal defects respectively; four defects were not detected on EC summed image (22,3%) possibly because the only A/P views, the lower counts and the higher pixel size of dynamic acquisition.

**Conclusion:** DMSA-scan is the gold standard test for parenchymal abnormalities and for the DRF. Ours preliminary results show that EC images provide an accurate DRF calculation and detection of scars in children with reflux. The absorbed radiation dose for EC is lower than DMSA and represents an advantage in pediatrics. Further studies are needed to determine if EC scans could be used alone to assess cortical lesions and DRF in selected cases. More cases are necessary to obtain a more accurate dosimetric evaluation.

For the study, the approval of the local ethics committee was not necessary as both the radiopharmaceuticals, (Tc 99m L-ethylenedicysteine and s Tc 99m DMSa) are regularly registered for diagnostic use in Italy. In addition, the patients used in the evaluation were chosen from all those who performed the diagnostic test for clinical reasons and requested from them reference specialist urologist.

**References**

1. Vallabhajousula S. et al. j of Nucl Med vol 30. N5 May 1989

2. Domingues F.C. et al Int Braz J urol. 2006;32:405-0

## PP09 A New Program for the Optimisation of Mo99-Tc99m Generator Selection

### W. H. Thomson

#### Physics and Nuclear Medicine, City Hospital, Birmingham UK

**Aim:** Generator costs are expected to rise significantly in the next few years. This is due to issues around reactor closures and future supplies being based on full cost recovery. Optimising the regular supply of Mo99 generators is almost impossible to do by trial and error, particularly for larger radiopharmacies getting two generators a week. Even single generator use departments face problems since all the manufacturers have different delivery schedules and reference activities. I developed an EXCEL program 6 years ago which analysed all generators on the UK market to match a departments requirements. This program has had to be fully re-written for various factors described.

**Methods:** The generator range on the UK/EC market has significantly expanded. In 2012 there were *N* = 112 different generators available (from 3 manufacturers), but in 2017 there are now *N* = 303 different generators,(from 4 manufacturers).. When both single generator and 2 generator usage is considered the number of possible options of generator supply are [N*(N-1)]/2+2N. So the number of combinations has risen from 6,440 to 46,359. The old program could not accommodate this number of generators. Also, the calculation algorithms of the old program with this increase in numbers would take over 40minutes.

**Results:** The program has been completely rewritten, with the following -

- Manufacturers’ generators can occupy separate worksheets.
- These individual worksheets are now separately selectable (included or excluded)
- Calculation of each generator Tc99m activity is automatic (this significantly aids data entry)
- Generators are pre-populated for major manufacturers so only the local costs are entered.
- 7day or 14day generator use is user selectable.
- Results are sorted in order of increasing cost, so usually only the first batch of results need to be reviewed.

However the major change is the processing algorithm, which now takes 840,000 combinations and take only 6seconds.

**Conclusion:** The program will be described (and possibly presented). It is impossible to maximise the supply using trial and error, particularly for 2 generators a week, and there is a potential for cost savings to be made from optimising the generator supply.

**Reference**

1. W. H. Thomson et al. “EXCEL programs to optimise the cost and choice of generator schedules for radiopharmacies and to provide daily Tc99m activity information”. presented at the 2012 “Radioactive Isotopes in Molecular Imaging - 30th International Austrian winter symposium”

## PP10 Interdisciplinary preoperative management of thyroid patients and thyroid cancer incidence

### M. Kudlacek^1^, M. Karik^2^, C. Farhan^1^, H. Rieger^3^, W. Pokieser^3^, K. Glaser^2^, S. Mirzaei^1^

#### ^1^Institute of Nuclear Medicine with PET-Center, Wilhelminenspital, Vienna, Austria; ^2^Department of Viceral and General Surgery, Wilhelminenspital, Vienna, Austria; ^3^Institute of Pathology and Microbiology, Wilhelminenspital, Vienna, Austria

##### **Correspondence:** M. Kudlacek

**Aim:** The increase in incidence of thyroid cancer during the last decades (about 2% of new cancer cases in Austria) without concomitant rise in mortality may reflect the growing detection of indolent forms of thyroid cancer. Our aim was to analyze the cancer statistics of our patients who underwent surgery in the time period of 2012-2016..

**Methods:** In the time period of 2012-2016 a total number of 636 patients (473 f, 166 m, age range 12-92y, median age 52 + 13,83y) were sent after decision making in thyroid board, consisted of nuclear medicine, surgery, pathology and ear-nose-throat (ENT) specialists. All surgical specimens were examined at the department for pathology in our hospital. Preoperative examination included sonography of thyroid gland and lymphatic tissue, scintigraphy, fine needle aspiration cytology in case of clinically suspect nodules, otolaryngological examination and laboratory analysis including Calcitonin if nodules were present.

**Results:** The temporal analysis of our patients in the time period of 2012-2016 revealed 135 cancer cases with following histological diagnosis:

54 PTC, 13FTC, 7 MTC, 1 anaplastic cancer, 1 metastasis of a renal cancer. 59 were staged as pT1a (6 pT1a(m) > 5mm, 1 tall-cell variant > 5mm with lymph vessel involvement and 2 with lymph node metastasis) and 26 patients with > pT1a had LN metastasis, and 2 showed distant metastasis at the time of diagnosis.

The annual incidence (%) of thyroid cancer (TC) was as in the following Table 2.

**Conclusion:** We observed a high cancer incidence of about 21% in our patients compared to the low rate of 2% thyroid cancer patients of new cancer cases in Austria. To our opinion a stringent patient selection in the interdisciplinary preoperative management of thyroid patients according to guidelines is the decisive factor for this high rate of thyroid cancer in our patients.

**References**

1. Austrian Thyroid Association: Modifizierte ATA-Empfehlungen zur Abklärung von Schilddrüsenknoten und Behandlung des differenzierten Schilddrüsenkarzinoms

**Table 2 (abstract PP10).** See text for description

Year	(f)	(m)	Total
2012	17	6	14
2013	20	17	19
2014	18	31	21
2015	22	9	19
2016	31	27	30

## PP11 Ultrasound criteria for risk stratification of thyroid nodules in the previously iodine deficient area of Austria. A single centre, retrospective analysis

### V. Petz^1^, C. Tugendsam^1^, W. Buchinger^2^, B. Schmoll-Hauer^1,3^, I. P. Schenk^1,4^, K. Rudolph^1^, M. Krebs^1,5^, G. Zettinig^1^

#### ^1^Schilddruesenpraxis Josefstadt, Vienna, Austria; ^2^Schilddrueseninstitut Gleisdorf, Gleisdorf, Austria; ^3^Department of Nuclear Medicine, Krankenanstalt Rudolfstiftung, Vienna, Austria; ^4^Department of Nuclear Medicine, Sozialmedizinisches Zentrum Hietzing, Vienna, Austria; ^5^Clinical Division of Endocrinology, Department of Medicine III, Medical University of Vienna, Vienna, Austria

**Aim:** We aimed to study the validity of six published ultrasound criteria for risk stratification of thyroid nodules in the former severely iodine deficient population of Austria.

**Methods:** Retrospective, single centre, observer blinded study design. All patients with a history of thyroidectomy due to nodules seen in the centre between 2004 and 2014 with preoperative in-house sonography and documented postoperative histology were analyzed (n=195). A board of five experienced thyroidologists evaluated the images of 45 papillary carcinomas, 8 follicular carcinomas, and 142 benign nodules regarding the following criteria: mild hypoechogenicity, marked hypoechogenicity, microlobulated or irregular margins, microcalcifications, taller than wide shape, missing thin halo.

**Results:** All criteria but mild hypoechogenicity were significantly more frequent in thyroid cancer than in benign nodules. The number of positive criteria was significantly higher in cancer (2.79±1.35) than in benign nodules (1.73±1.18; p<0.001). Thus, with a cut-off of two or more positive criteria, a sensitivity of 85% and a specificity of 45% were reached to predict malignancy in this sample of thyroid nodules. As expected, the findings were even more pronounced in papillary cancer only (2.98±1.32 vs. 1.73±1.18, p<0.001). The six ultrasound criteria could not identify follicular cancer.

**Conclusion:** Our findings support the recently published EU-TIRADS score. Apart from mild hypoechogenicity, the analyzed ultrasound criteria can be applied for risk stratification of thyroid nodules in the previously severely iodine deficient population of Austria.

## PP12 Evaluation of cerebral P-glycoprotein function in an Alzheimer’s disease mouse model with positron emission tomography

### V. Zoufal^1^, T. Wanek^1^, M. Krohn^2^, S. Mairinger^1^, J. Stanek^1,3^, M. Sauberer^1^, T. Filip^1^, J. Pahnke^2^, O. Langer^1,3^

#### ^1^Center for Health & Bioresources, AIT Austrian Institute of Technology GmbH, Seibersdorf, Austria; ^2^Department of Neuro-/Pathology, University of Oslo (UiO) and Oslo University Hospital (OUS), Oslo, Norway; ^3^Department of Clinical Pharmacology, Medical University of Vienna, Vienna, Austria

##### **Correspondence:** V. Zoufal

**Aim:** P-glycoprotein (Pgp), a membrane transporter expressed at the blood-brain barrier (BBB), may contribute to clearance of beta-amyloid (Aß) from the brain [1, 2]. Positron emission tomography (PET) with the Pgp substrate (R)-[11C]verapamil ([11C]VPM) has shown that cerebral Pgp function is reduced in Alzheimer’s disease (AD) patients and during healthy ageing [3, 4]. Transgenic mouse models are commonly used in AD research. It is currently not known if [11C]VPM PET possesses adequate sensitivity to detect a moderate reduction in Pgp function at the BBB as it occurs in AD mouse models. In the present study, we employed a novel partial Pgp inhibition protocol using tariquidar [4] to assess Pgp function with [11C]VPM PET in one commonly employed AD mouse model (APPPS1 mice) and in age-matched wild-type mice.

**Methods:** Female C57BL/6N wild-type and APPPS1 mice aged 50 days and 200 days underwent dynamic [11C]VPM PET scans after pre-treatment with vehicle or the Pgp inhibitor tariquidar at a dose of 4 mg/kg leading to partial Pgp inhibition at the BBB (n = 4 - 6). In addition, heterozygous Pgp knockout mice (Abcb1a/b(+/-)) were studied as a model of 50% reduction in Pgp density at the BBB. At the end of the PET scan a venous blood sample was collected by retro-orbital puncture. Brain uptake of [11C]VPM was expressed as the brain-to-plasma ratio of radioactivity in the last PET frame (Kp,brain). Plasma was analyzed by radio-TLC for radiolabeled metabolites of [11C]VPM. Immunohistochemical (IHC) staining was performed to visualize the spatial distribution of Pgp in the brain.

**Results:** In PET scans without Pgp inhibition, Kp,brain values of [11C]VPM were not significantly different between all mouse groups. Administration of tariquidar led to increases in Kp,brain values, which differed among groups with the following rank order: wild-type 50 days: +23 ± 10%, APPPS1 50 days: +31 ± 14%, wild-type 200 days: +31 ± 11%, APPPS1 200 days: +39 ± 23%, Abcb1a/b(+/-): +59 ± 6%. After tariquidar administration, Kp,brain values were significantly higher in wild-type mice aged 200 days than 50 days (1.02 ± 0.10 vs. 0.90 ± 0.11, p = 0.04, Student’s t-test). In addition, higher Kp,brain values were detected in APPPS1 mice aged 50 days compared to wild-type mice aged 50 days (1.09 ± 0.07 vs. 0.90 ± 0.11, p = 0.01). In Abcb1a/b(+/-) mice, Kp,brain values after tariquidar administration (1.32 ± 0.11) were significantly higher than in wild-type and APPPS1 mice from both age groups. No differences in the percentage of radiolabeled metabolites of [11C]VPM in plasma were observed between wild-type and APPPS1 mice. IHC analysis of brain slices confirmed reduced Pgp expression in brain capillaries of APPPS1 mice as compared with wild-type mice.

**Conclusion:** Our data confirm previous findings that [11C]VPM PET without inhibitor administration lacks the sensitivity to detect moderate changes in Pgp function at the BBB [4]. We obtained evidence that [11C]VPM PET in combination with partial Pgp inhibition with tariquidar can be used to detect age- and AD-related reductions in Pgp function at the BBB of mice. The employed PET protocol may prove useful in future studies evaluating different therapeutic approaches to restore Pgp function at the BBB.

**References**

1. Mawuenyega KG, Sigurdson W, Ovod V, Munsell L, Kasten T, Morris JC, et al. Decreased clearance of CNS beta-amyloid in Alzheimer's disease. Science 2010;330:1774.

2. Cirrito JR, Deane R, Fagan AM, Spinner ML, Parsadanian M, Finn MB, et al. P-glycoprotein deficiency at the blood-brain barrier increases amyloid-beta deposition in an Alzheimer disease mouse model. J Clin Invest 2005;115:3285-90.

3. Deo AK, Borson S, Link JM, Domino K, Eary JF, Ke B, et al. Activity of P-glycoprotein, a beta-amyloid transporter at the blood-brain barrier, is compromised in patients with mild Alzheimer disease. J Nucl Med 2014;55:1106-11.

4. Bauer M, Wulkersdorfer B, Karch R, Philippe C, Jager W, Stanek J, et al. Effect of P-glycoprotein inhibition at the blood-brain barrier on brain distribution of (R)-[11 C]verapamil in elderly versus young subjects. Br J Clin Pharmacol 2017;83:1991-9.

## PP13 The curious fever: The diagnostic value of 18F-FDG in FUO in a large single-center retrospective study on 300 patients

### F. Weitzer, B. Pernthaler, S. Salamon, R. Aigner

#### Meduni Graz, Univ. Klinik für Radiologie, Abteilung für Nuklearmedizin, Graz, Austria

##### **Correspondence:** F. Weitzer

**Aim:** 18F-FDG PET/CT has been described as a helpful diagnostic tool in FUO (fever of unknown origin) when other radiological or laboratory methods fail to explain the fever origin. We present preliminary results from a large single-center retrospective study on the diagnostic value of 18F-FDG PET/CT in FUO.

**Methods:** We included 143 patients (65 male 45%; 78 female 55%) who presented with a history of FUO. Microbiological and histological findings, patient follow-up and further treatment were retrospectively correlated to PET/CT findings.

**Results:** In 109 (76.2%) cases, PET/CT findings correlated positively with the final diagnosis, while in 17 (11.9%) cases the PET/CT findings only partially correlated with the final diagnosis. In 17 (11.9%) cases PET/CT findings were false-positive. In 56 (39.2%) cases PET/CT showed no focus.

In in 43 cases (30.1%) fever was caused by various infectious diseases; in 22 cases (15.4%) fever was a sign of malignoma; in 21 cases (14.7%), fever was caused by autoimmune or rheumatic diseases; in 6 cases (4.2%) miscellanceous diseases were reported. In 28 cases (19.6%) no underlying disease could be found.

In 23 (16.1%) cases a plausible cause for fever was previously known, PET/CT was performed to outrule another infectious or malignant source, in this cases PET/CT showed no other focus or signs of malignancy.

**Conclusion:** 18F-FDG PET/CT is the gold standard in FUO diagnostics in nuclear medicine, particularly when other radiological and laboratory methods fail to provide an explanation for FUO. Our study delivers important data for the use of 18F-FDG-PET/CT in FUO, since there is still a lack of large studies to support this hypothesis.

**References**

1. Schönau V, Vogel K, Englbrecht M, Wacker J, Schmidt D, Manger B, et al. The value of 18F-FDG-PET/CT in identifying the cause of fever of unknown origin (FUO) and inflammation of unknown origin (IUO): data from a prospective study. Ann Rheum Dis. 2017 Sep 19. pii: annrheumdis-2017-211687. 10.1136/annrheumdis-2017-211687. [Epub ahead of print]

2. Bharucha T, Rutherford A, Skeoch S, Alavi A, Brown M, Galloway J; Diagnostic yield of FDG-PET/CT in fever of unknown origin: a systematic review, meta-analysis, and Delphi exercise. Clin Radiol. 2017 Sep;72(9):764-771. 10.1016/j.crad.2017.04.014. Epub 2017 Jun 7.

3. Besson FL, Chaumet-Riffaud P, Playe M, Noel N, Lambotte O, Goujard C, et al. Contribution of (18)F-FDG PET in the diagnostic assessment of fever of unknown origin (FUO): a stratification-based meta-analysis. Eur J Nucl Med Mol Imaging. 2016 Sep;43(10):1887-95. 10.1007/s00259-016-3377-6. Epub 2016 Apr 2. Review.

## PP14 18F-FDG PET/CT in the detection of an early infection of arteriovenous grafts in hemodialysis patients

### P. Koranda^1^, L. Henzlová^1^, M. Kamínek^1^, Mo. Váchalová^2^, P. Bachleda^2^

#### ^1^Department of Nuclear Medicine, University Hospital Olomouc and Palacky University, Olomouc, Czech Republic; ^2^Department of Vascular and Transplantation Surgery, University Hospital Olomouc and Palacky University, Olomouc, Czech Republic

##### **Correspondence:** P. Koranda

**Aim:** For patients on chronic hemodialysis (HD) treatment, vascular access is required. Insertion of arteriovenous graft (AVG) is indicated in patients with a failed arteriovenous fistula. The aim of this study was to evaluate 18F-FDG PET/CT in detection of early AVG infections. Early treatment of AVG infection is important, because an advanced prosthetic infection usually leads to removal of the prosthesis.

**Methods:** A total of 51 AVGs was evaluated. One-year monitoring was completed in 30 AVGs. 18F-FDG PET/CT was performed at intervals of 10, 20–30, and 40–50 weeks after AVG insertion. Increased accumulation of FDG and WBC in the prosthesis was classified as focal or diffuse. An agreement between the 18F-FDG PET/CT and “reference” parameters (clinical local status, CRP, and microbiological evaluation of a swab sampled from the HD cannula immediately after the HD procedure) was evaluated using the Gwet’s coefficient AC1 and McNemar test for symmetry.

**Results:** At 10 weeks since AVG implantation the AC1 values were: focal 18F-FDG accumulation - clinical status AC1 0.693, CRP AC1 0.605, cannula microbiology AC1 0.518; focal and diffuse 18F-FDG accumulation - clinical status AC1 0.617, CRP AC1 0.695 (but McNemar 0.039), cannula microbiology AC1 0.628; diffuse 18F-FDG accumulation - clinical status AC1 0.079, CRP AC1 0.167, cannula microbiology AC1 0.255. At 20 to 30 weeks after AVG implantation: focal 18F-FDG accumulation - clinical status AC1 0.656, CRP AC1 0.570, cannula microbiology AC1 0.409; focal and diffuse 18F-FDG accumulation - clinical status AC1 0.596, CRP AC1 0.669, cannula microbiology AC1 0.518 (McNemar 0.012),; diffuse 18F-FDG accumulation - clinical status AC1 0.073 (McNemar 0.041), CRP AC1 0.166, (McNemar 0.031), microbiology AC1 0.076. Between 40 and 50 weeks since AVG implantation: focal 18F-FDG accumulation - clinical status AC1 0.524 (McNemar 0.039), CRP AC1 0.456, cannula microbiology AC1 0.569; focal and diffuse 18F-FDG accumulation - clinical status AC1 0.673, CRP AC1 0.710, cannula microbiology AC1 0.720; diffuse 18F-FDG accumulation - clinical status AC1 0.347 (McNemar 0.039), CRP AC1 0.385, cannula microbiology AC1 0.371.

**Conclusion:** The study results show that focal accumulation of 18F-FDG (in combination with diffuse uptake or solely) can be considered as a sign of early AVG infection in HD patients. This fact is consistent with the results of similar 18F-FDG PET/CT studies concerning infections of common AVG grafts (1, 2). 18F-FDG PET/CT can serve as a supportive parameter indicating the need for antibiotic therapy in early infection of AVG in HD patients.

**References**

1. Berger P, Vaartjes I, Scholtens A, et al. Differential FDG-PET uptake pPatterns in uninfected and infected Central Prosthetic Vascular Grafts. Eur J Vasc Endovasc Surg. 2015; 50(3):376-83.

2. Spacek M, Belohlavek O, Votrubova J, et al. Diagnostics of "non-acute" vascular prosthesis infection using 18F-FDG PET/CT: our experience with 96 prostheses. Eur J Nucl Med Mol Imaging. 2009; 36(5):850-8.

## PP15 89Zr-Siderophore-Affibody conjugates for imaging EGFR expression

### D. Summer^1^, J. Garousi^2^, M. Oroujeni^2^, B. Mitran^3^, K. G. Andersson^4^, A. Vorobyeva^2^, J.n Löfblom^4^, A.Orlova^3^, V. Tolmachev^2^, C. Decristoforo^1^

#### ^1^Department of Nuclear Medicine, Medical University Innsbruck, Anichstrasse 35, A-6020 Innsbruck, Austria; ^2^Institute of Immunology, Genetic and Pathology, Uppsala University, SE-75185 Uppsala, Sweden; ^3^Division of Molecular Imaging, Department of Medicinal Chemistry, Uppsala University, SE-751 83 Uppsala, Sweden; ^4^Division of Protein Technology, KTH Royal Institute of Technology, SE-10691 Stockholm, Sweden

##### **Correspondence:** C. Decristoforo

**Aim:** Zirconium-89 has gained great interest for PET, when imaging at late time points is required. Desferrioxamine B (DFO), is mostly used for this radionuclide as bifunctional chelator (BFC) and we recently reported on fusarinine C (FSC) with similar zirconium-89 complexing properties but potentially higher stability related to its cyclic structure. This study reports on the comparison of FSC and DFO as BFCs for 89Zr labelling of the affibody ZEGFR:2377 targeting Epidermal Growth Factor Receptors (EGFR).

**Methods:** FSC-ZEGFR:2377 and DFO-ZEGFR:2377 were evaluated regarding labeling, in vitro stability, specificity, cell uptake, receptor affinity, biodistribution and microPET-CT imaging.

**Results:** Both conjugates showed increased labelling yields at elevated temperature (85°C). Both conjugates revealed remarkable specificity, affinity and slow cell-line dependent internalisation. Labeling at 85°C showed comparable results in A431 tumor xenografted mice with minor differences regarding blood clearance, tumor and liver uptake but clear improvement as compared to 89Zr-DFO-ZEGFR:2377, labeled at room temperature, which was confirmed by MicroPET-CT imaging.

**Conclusion:** We were able to show that FSC is a suitable alternative to DFO for labeling of biomolecules with zirconium-89. Furthermore our findings indicate that 89Zr- labeling of DFO conjugates at higher temperature reduces off-chelate binding leading to significantly improved tumor-to-organ ratios and therefore enhancing image contrast.

## PP16 TAFC Modifications

### P. Kaeopookum^1,4^, D. Summer^1^, T. Orasch^2^, B. Lechner^2^, M. Petrik^3^, Z. Novy^3^, C. Rangger^1^, H. Haas^2^, C. Decristoforo^1^

#### ^1^Department of Nuclear Medicine, Medical University Innsbruck, Innsbruck, Austria; ^2^Division of Molecular Biology, Biocenter, Medical University Innsbruck, Innsbruck, Austria; ^3^Faculty of Medicine and Dentistry, Institute of Molecular and Translation Medicine, Palacky University, Olomouc, Czech Republic; ^4^Research and Development Division, Thailand Institute of Nuclear Technology, Nakhonnayok, Thailand

##### **Correspondence:** C. Decristoforo

**Aim:** Aspergillus fumigatus produces the siderphore triacetylfusarinine C (TAFC) for iron acquisition and is essential for its virulence, therefore a specific marker for invasive aspergillosis. [68Ga]TAFC exhibited excellent targeting properties in A. fumigatus infection model. We aimed to modify TAFC to investigate the influence of introduced substitutes on preservation of TAFC characteristics in vitro and in vivo.

**Methods:** Various TAFC modifications with various substitution (different carbon chain length acyl compounds as well as charge substitutents) were prepared and labelled with 68Ga. Stability, log P and Protein binding was assessed. Uptake and growth studies in Aspergillus species expressing the specific TAFC Transporter (MirB) were performed. Normal biodistribution and μPET Imaging of selected compounds was performed.

**Results:** In vitro uptake studies using A. fumigatus showed the recognition of MirB to monosubstituted TAFC whereas uptake assays of MirB-possesing- and wildtype-A. terreus confirmed the specificity to TAFC of MirB transporter. Llipophilicities as expressed in logD were -0.38 to -3.80. One selected compound, [68Ga]DABuFC displayed low protein binding and was stable in PBS and serum and revealed comparable biodistribution behaviors and image contrast by PET/CT compared to [68Ga]TAFC.

**Conclusion:** Our studies show the possibility to modify TAFC without losing its properties and recognition of MirB. Introducing functionalities such as fluorescent dyes or anti-infection moieties opens new ways for theranostics of infection diseases.

